# Specificity of Small *c*-Type Cytochromes in Anaerobic Ammonium Oxidation

**DOI:** 10.1021/acsomega.1c02275

**Published:** 2021-08-09

**Authors:** Mohd. Akram, Josephine Bock, Andreas Dietl, Thomas R.M. Barends

**Affiliations:** Department of Biomolecular Mechanisms, Max Planck Institute for Medical Research, Jahnstrasse 29, D-69120 Heidelberg, Germany

## Abstract

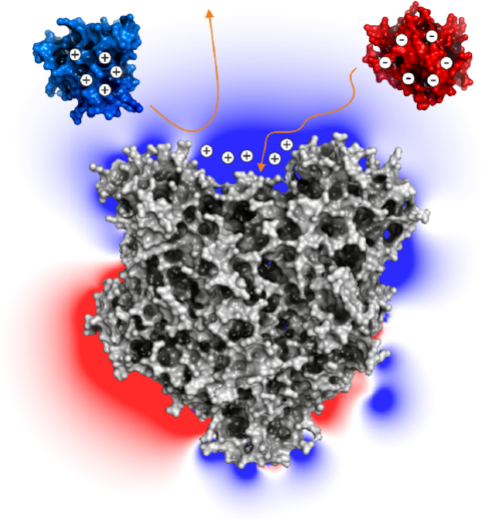

Anaerobic ammonium oxidation (anammox) is a bacterial process in which ammonium and nitrite are combined into dinitrogen gas and water, yielding energy for the cell. This process relies on a series of redox reactions catalyzed by a set of enzymes, with electrons being shuttled to and from these enzymes, likely by small cytochrome *c* proteins. For this system to work productively, these electron carriers require a degree of specificity toward the various possible redox partners they encounter in the cell. Here, we compare two cytochrome *c* proteins from the anammox model organism *Kuenenia stuttgartiensis*. We show that they are highly homologous, are expressed at comparable levels, share the same fold, and display highly similar redox potentials, yet one of them accepts electrons from the metabolic enzyme hydroxylamine oxidase (HAO) efficiently, whereas the other does not. An analysis of the crystal structures supplemented by Monte Carlo simulations of the transient redox interactions suggests that this difference is at least partly due to the electrostatic field surrounding the proteins, illustrating one way in which the electron carriers in anammox could attain the required specificity. Moreover, the simulations suggest a different “outlet” for electrons on HAO than has traditionally been assumed.

## Introduction

**Figure 1:**
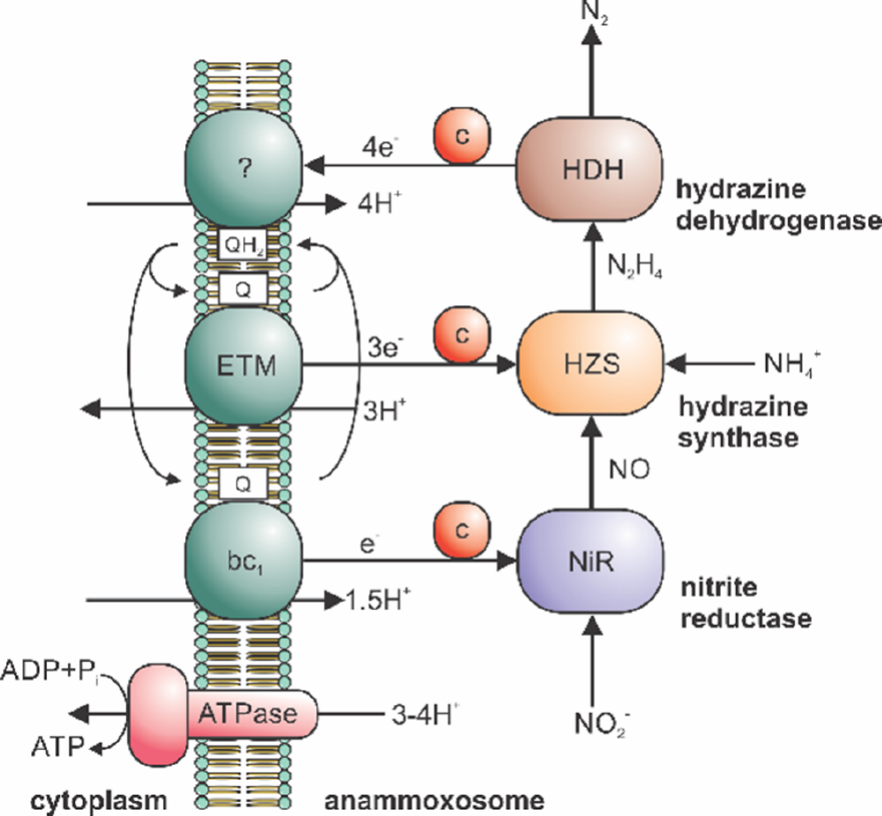
Overview of the central anammox metabolism. An electron transport chain located in the membrane of the anammoxosome is driven by a set of redox reactions catalyzed by NiR, HZS, and HDH. As these are soluble enzymes distributed across the interior of the anammoxosome, a transport system is required to shuttle electrons between these enzymes and the membrane, which is proposed to involve *c*-type cytochromes (red, labeled “c”).

Anaerobic ammonium oxidation (anammox) is a bacterial process in which ammonium (NH_4_^+^) and nitrite (NO_2_^–^) are converted into dinitrogen gas (N_2_) and water, yielding energy for the cell.^1^ The process takes place in a specialized, membrane-enclosed cellular compartment, the anammoxosome,^2^ which contains the redox-active proteins at the heart of the process (Figure 1). The first step is the one-electron reduction of NO_2_^–^ to nitric oxide (NO) by a nitrite reductase (NiR).^3−6^ The resulting NO is then combined with NH_4_^+^ to yield the extremely reactive and unusual intermediate hydrazine (N_2_H_4_) by the unique hydrazine synthase (HZS)^7^ in a process that takes up a further three electrons. This reaction likely proceeds via hydroxylamine (NH_2_OH) as an intermediate. It is assumed that any hydroxylamine escaping from HZS is converted back into NO by the octaheme *c*-type cytochrome hydroxylamine oxidase (HAO),^8,9^ releasing three electrons. Finally, the hydrazine is oxidized to N_2_ by hydrazine dehydrogenase (HDH),^9,10^ releasing four electrons at an extremely low redox potential of −750 mV. Together, these redox reactions fuel an electron transport chain that generates a proton gradient across the anammoxosomal membrane. This proton gradient, in turn, is used to drive ATP synthesis.^1^ Strikingly, NiR, HZS, HAO, and HDH are all soluble proteins distributed throughout the anammoxosome,^11^ yet the flow of electrons set up by their redox reactions must necessarily pass through the membrane to allow proton translocation. Thus, an efficient electron transport system is required to shuttle electrons between the membrane on the one hand and the various soluble redox proteins on the other hand. Moreover, this system has to be highly specific, as e.g. the low-potential electrons released by HDH should be transported at least preferentially to the membrane, rather than being shuttled to, for instance, NiR or HZS, since this would result in an effective short-circuit of the system.^10^

As anammox bacteria express a large number of small *c*-type cytochromes, these have been proposed to perform the shuttling of electrons between the various parts of the metabolic machinery. However, few of these have been characterized and little is known about the precise role of the various representatives of this class in the anammox metabolism, including how they recognize their binding partners. Here, we present the structures and biochemical characterization of two of these proteins from the anammox model organism *Kuenenia stuttgartiensis*: Kustc0562 and highly homologous Kustc0563, which show relatively high transcription levels (Figure S1). Indeed, Kustc0563 was the first protein from an anammox organism to be purified from biomass^12^ and also the first to be heterologously expressed and characterized.^13^ We show that the two proteins are highly similar structurally and electrochemically yet find that Kustc0563 is able to efficiently accept electrons from *K. stuttgartiensis* HAO (also known as Kustc1061) in hydroxylamine oxidation assays, whereas Kustc0562 does not do so. Moreover, Monte Carlo simulations of the electrostatic interactions indicate that a disparity in their charge distributions contributes to this difference. Moreover, the simulations suggest a different “outlet” for electrons from HAO when interacting with Kustc0563 than that has traditionally been assumed for this class of proteins.

## Results

### Kustc0562 and Kustc0563 have Very Similar Structures

**Figure 2:**
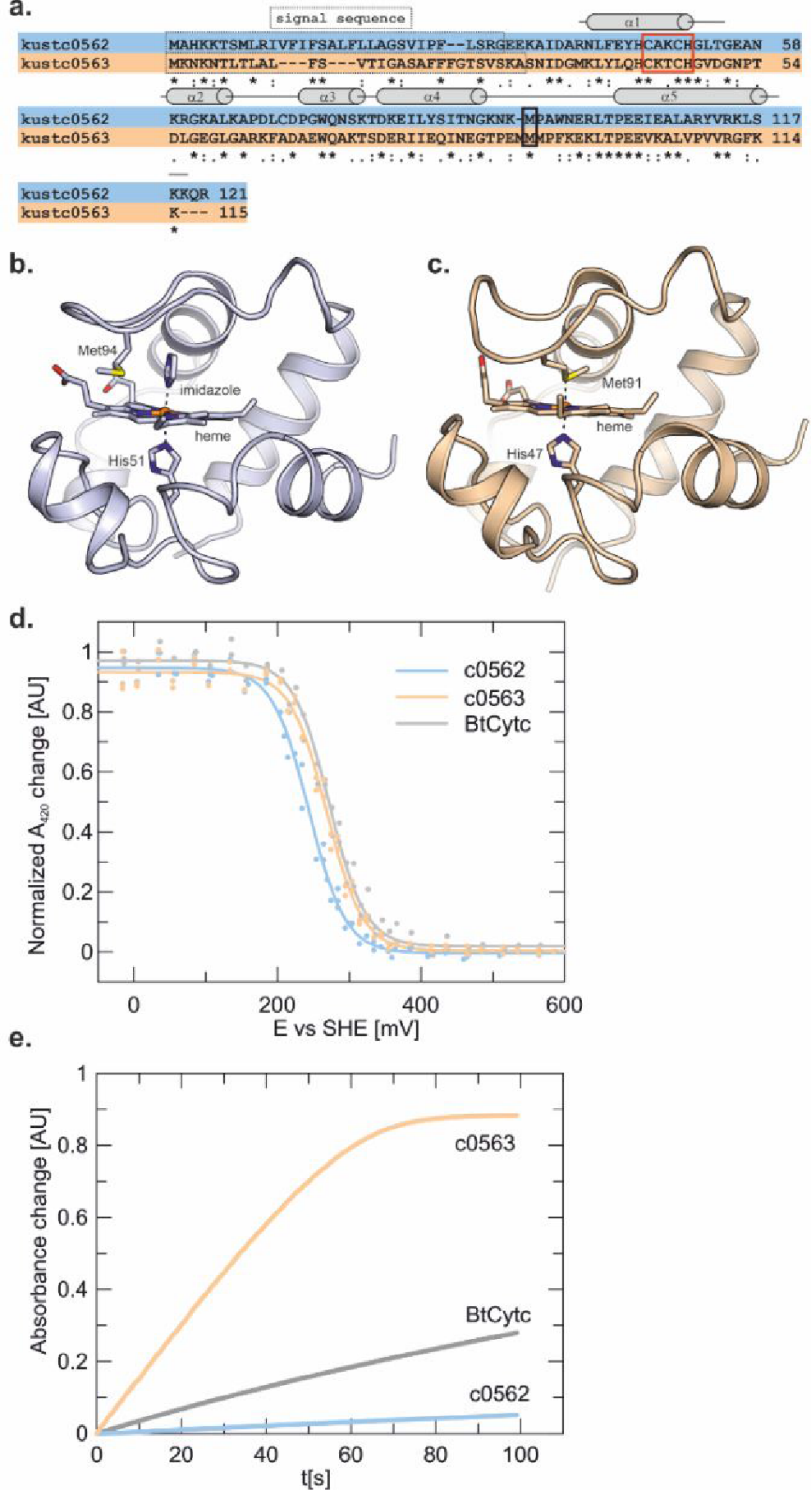
(a) Alignment of the sequences of Kustc0562 and Kustc0563. The signal sequence and the secondary structure elements are shown above the sequence. The heme binding motif and the distal methionine are indicated by the red and black boxes, respectively. (b) Crystal structure of Kustc0562. The protein is shown as a cartoon, with the heme and the iron-coordinating residues shown as sticks. The cysteine residues bound covalently to the heme were omitted for clarity. (c) Crystal structure of Kustc0563 presented as in panel (b). (d) Spectroelectrochemistry of Kustc0562, Kustc0563, and bovine (*Bos taurus*) cytochrome *c* (BtCytc). The normalized heme absorbance at 420 nm is shown as a function of the applied potential vs SHE. (e) Progression curves of hydroxylamine oxidation (100 μM) catalyzed by KsHAO (1.2 μg mL^–1^) using 50 μM either Kustc0562, Kustc0563, or BtCytc as the electron acceptor. The absorbance change at 550 nm is plotted against time starting from substrate addition.

Kustc0562 and Kustc0563 share 37% sequence identity after cleavage of the signal sequence (Figure 2a) and display a typical type-I cytochrome *c* fold (Figure 2b,c). Their crystal structures can be superimposed to an RMSD of 1.2 Å for 81 Cα atoms. In the crystal structure of Kustc0563, the heme iron is proximally coordinated by His47 and distally by Met91; in Kustc0562, the iron is proximally coordinated by the homologous His51, but the distal ligand is an imidazole molecule, presumably from the crystallization solution. Met94, the residue in Kustc0562 homologous to the distal Met in Kustc0563, has moved toward the side of the heme with the propionate groups to make space for imidazole, and a part of the loop containing this residue shows a different conformation than that in Kustc0563. Indeed, when 100 mM imidazole was added to oxidized Kustc0562, the UV–vis spectrum showed a shift of the Soret band from 411 to 406 nm, which was accompanied by the disappearance of a weak charge-transfer band at 690 nm, indicative of methionine coordination to the heme iron^14^ (Figure S2a). When imidazole was removed by buffer exchange, the spectral features of oxidized Kustc0562 were restored. The same effect was observed for Kustc0563 (Figure S2b). In both proteins, the heme surroundings are predominantly hydrophobic.

### Kustc0562 and Kustc0563 have Very Similar Redox Potentials

Spectropotentiometry (Figure 2d and S3) revealed that Kustc0562 and Kustc0563 have redox potentials of +244 and +268 mV versus standard hydrogen electrode (SHE), respectively. These values are similar to the redox potential of bovine mitochondrial cytochrome *c* (BtCytc), which had earlier been reported to be +262 mV versus SHE^15^ and for which we find the value to be +272 mV versus SHE. Previously, Huston et al.^13^ had found a redox potential of +230 mV versus SHE for Kustc0563. These values are all very similar in light of the redox potential of the hydroxylamine/nitric oxide redox pair, which is −30 mV versus SHE.

### Kustc0563 Is Able to Accept Electrons from KsHAO, but the Highly Similar Kustc0562 is Much Less So

We performed assays measuring the rate of hydroxylamine oxidation by KsHAO using Kustc0562, Kustc0563, and BtCytc as electron acceptors (Figure 2e and S4 and Table 1). The highest activity was measured using Kustc0563 with a *V*_max_ of about 6 times higher than that obtained with BtCytc. Strikingly, however, despite the structural and electrochemical similarities between Kustc0562 and Kustc0563, only marginal activity could be detected when Kustc0562 was used as the electron acceptor.

**Table 1:** Enzyme Kinetics of Hydroxylamine Oxidation by KsHAO with Various Electron Acceptors^a^

electron acceptor	*V*_max_ NH_2_OH [μmol min^–1^ mg^–1^]	*K*_m_ NH_2_OH [μM]	k_cat_ NH_2_OH [s^–1^] (KsHAO trimer)	k_cat_/K_m_ [μM^–1^ s^–1^]
Kustc0562	n.d.	n.d.	n.d.	n.d.
Kustc0563	15.8 ± 0.3	13.9 ± 0.7	48.4	3.5
BtCytc	2.7 ± 0.1	5.8 ± 0.7	8.4	1.4

a With Kustc0562, only marginal activity could be detected and no Michaelis–Menten parameters were determined.

### Monte Carlo Interaction Mapping Suggesting a Binding Interface between Kustc0563 and HAO

**Figure 3:**
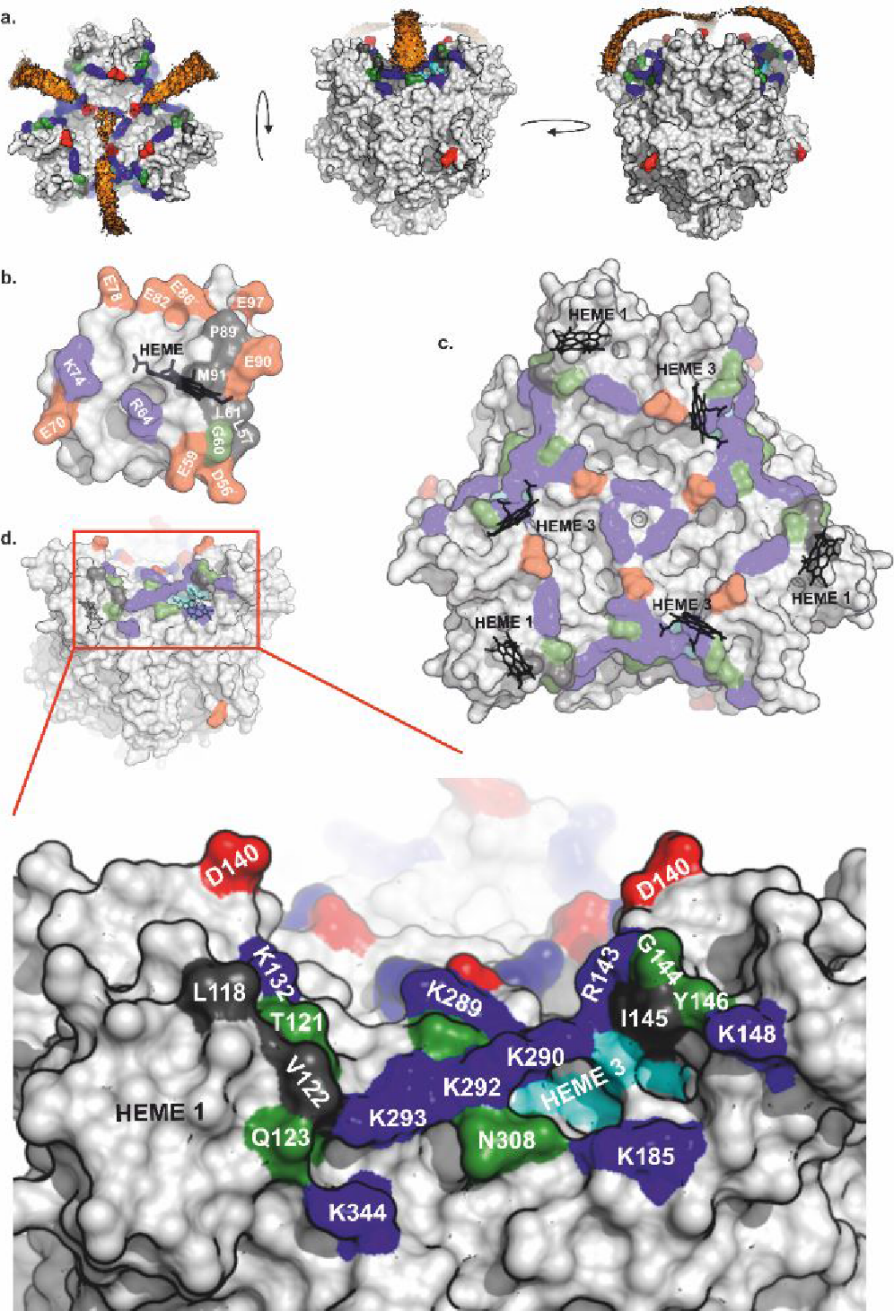
Results of the Monte Carlo mapping of the interaction between KsHAO and Kustc0563. (a) KsHAO is shown in three orientations, with the preferred positions of Kustc0563 during the Monte Carlo run shown in orange. (b) Predicted interaction surface on Kustc0563 (white), with the residues predicted to be involved in the interaction with KsHAO shown in color. The heme is shown as sticks, negatively charged residues are indicated in red, neutral residues are indicated in green, hydrophobic residues are indicated in gray, and positively charged residues are indicated in blue. (c) As (b) for the predicted interaction surface on KsHAO. (d) Closeup of the interaction surface on KsHAO using the same color coding as in panel (b).

To rationalize this difference in ability to accept electrons from HAO, we performed Monte Carlo interaction mapping to identify possible interaction sites between Kustc0563 and HAO using MCMAP 1.0.^16^ This software package allows a ligand protein to randomly move in the electrostatic field of a receptor molecule, resulting in an ensemble of mutual positions and orientations, thus taking the typically transient nature of interactions between redox-active proteins into account. The simulations predict a preferential interaction surface between Kustc0563 and KsHAO (Figure 3a). On Kustc0563, this involves a ring of negatively charged residues on the surface around the heme, in addition to several hydrophobic and two positively charged residues (Figure 3b). On the trimeric KsHAO, which is shaped like a tulip bulb with protrusions on three sides, the predicted interaction surface involves the concave area between two protrusions. This area comprises several positively charged residues, heme 3 of the proteins’ multiheme electron transport system (see below), as well as some neutral and hydrophobic residues (Figure 3c,d).

### Kustc0562 and Kustc0563 Have Very Different Electrostatics

**Figure 4:**
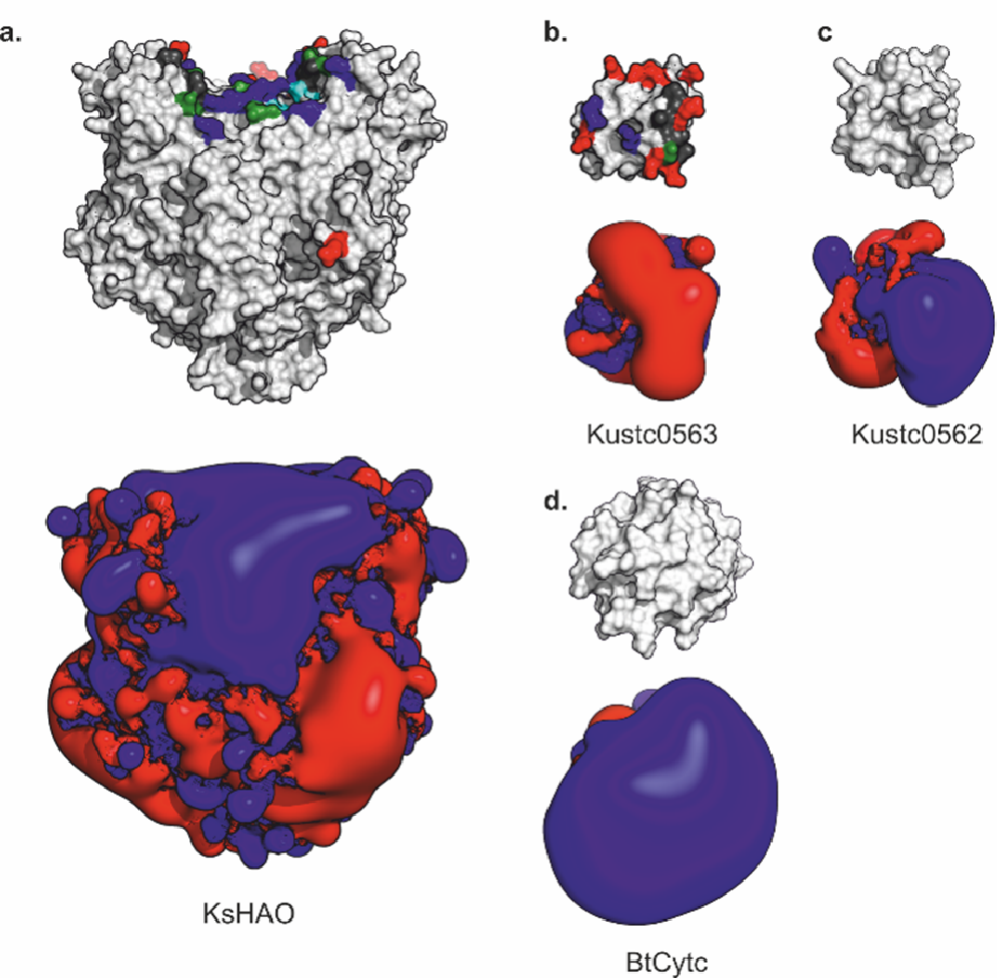
Electrostatics of KsHAO (a), Kustc0563 (b), Kustc0562 (c), and BtCytc (d). The top panels show the orientation of the protein, with the predicted interaction surfaces toward the viewer and color coded as in Figure 3. The orientation of Kustc0562 is the same as that of Kustc0563; BtCytc is oriented with the surface of the heme binding site oriented toward the viewer. The bottom panels show the +1 *k*_B_*T*/e (blue) and −1 *k*_B_*T*/e (red) isopotential surfaces as determined using APBS, assuming 20 mM phosphate buffer, as in the KsHAO activity assays.

Inspection of the electrostatics of Kustc0562, Kustc0563, and KsHAO offers an attractive explanation for the observed difference in the ability between Kustc0562 and Kustc0563 to serve as an electron acceptor for HAO in hydroxylamine oxidation reactions. The predicted interaction surface on HAO displays a pronounced positive charge, which would complement the equally pronounced negative charge on the predicted interaction surface on Kustc0563 (Figure 4). In contrast, despite the strong sequence similarity between the two proteins, the corresponding surface on Kustc0562 displays a pronounced positive charge, which would make interactions with the proposed binding site on the HAO surface electrostatically unfavorable.

## Discussion

Although several central anammox metabolic enzymes have been purified and characterized biochemically and/or structurally,^3−10^ little is known about the ways in which these proteins exchange electrons with other parts of the anammox metabolism. It has been suggested that small *c*-type cytochromes act as electron shuttles between the soluble redox enzymes of the anammox metabolism on the one hand, and the electron transport chain in the membrane on the other. NaxLS, a stable complex of two small cytochrome *c* proteins, was found to interact specifically with HZS in pulldown assays,^17^ and a putative cytochrome *c* binding site was identified on the HZS surface.^7^ Moreover, based on its hollow structure with small entrances to a central cavity, HDH was suggested to possess a specificity filter, allowing only small proteins to access binding sites for redox partners inside the internal cavity.^10^ However, few small cytochrome *c* proteins from anammox organisms have been studied in detail themselves. Kustc0563 was previously characterized electrochemically and spectroscopically by Huston and coworkers,^13^ who also predicted its α-helical nature by circular dichroism, and Akram et al.^17^ characterized NaxLS electrochemically, spectroscopically, and structurally. Hira et al. studied C0855, a small *c*-type cytochrome from *Jettenia caeni*, which displays a unique His/3,4-dihydroxyphenylalanine coordination of its heme iron.^18^

Here, we compare two small *c*-type cytochromes from *K. stuttgartiensis* in terms of their structure, electrochemistry, and ability to serve as electron acceptors for KsHAO.

The structures of Kustc0562 and Kustc0563 are highly similar, the main difference in the crystal structures being the presence of an axial imidazole ligand in the Kustc0562 structure, with an Fe–N distance of 2.1 Å. Given the high concentration of imidazole (100 mM) in the crystallization experiment, we believe this to be a crystallization artifact in which the “true” distal ligand, Met94, is displaced by imidazole. Indeed, when oxidized Kustc0562 was exposed in solution to the same concentration of imidazole as used in crystallization, a shift of the Soret band maximum from 411 to 406 nm accompanied by the disappearance of a charge-transfer band at 690 nm indicated a ligand exchange of methionine to imidazole.^14^ These spectral changes were reversed upon removal of imidazole. Similar results were obtained when Kustc0563 was treated with imidazole, but this protein could be crystallized without it.

Michaelis–Menten kinetics of hydroxylamine oxidation by KsHAO using BtCytc as the electron acceptor results in parameters (*k*_cat_ = 8.4 s^–1^, *K*_m_ = 5.8 μM) similar to the values reported previously^8^ (*k*_cat_ = 15 s^–1^, *K*_m_ = 4.4 μM). Importantly, however, the comparison of BtCytc with Kustc0563 shows that the latter protein supports hydroxylamine oxidation by KsHAO at a rate that is approximately 6 times higher (*k*_cat_ = 48.4 s^–1^, *K*_m_ = 13.9 μM).

Moreover, Kustc0562 supported KsHAO activity only at marginal rates under identical circumstances. The striking contrast in electrostatics around the interaction sites on Kustc0562 and Kustc0563 provides an attractive explanation for this difference. Like Kustc0562, BtCytc also displays a pronounced positive surface electrostatic charge around the heme access site^19,20^ (Figure 4d) and, as expected, given this, BtCytc is indeed a less efficient electron acceptor than Kustc0563 in HAO-catalyzed hydroxylamine oxidation. However, the fact that BtCytc is still able to accept electrons from HAO at a higher rate than Kustc0562 shows that simple electrostatics alone do not fully explain the differing abilities of various proteins to act as an electron acceptor in these reactions, and possibly, BtCytc interacts with KsHAO via a different interaction site than Kustc0563. Nevertheless, the differences in electrostatics between Kustc0562 and Kustc0563 do highlight a mechanism in which the much-needed specificity of electron transport proteins toward their redox partners may at least in part be conferred.

Moreover, it is important to note that the results presented here do not necessarily mean that Kustc0563 is a physiological redox partner for KsHAO. In *Nitrosomonas europaea*, the highly homologous hydroxylamine oxidoreductase (NeHAO) transfers the electrons derived from hydroxylamine oxidation to a dedicated tetraheme protein, cytochrome c554.^21,22^ The genome of *K. stuttgartiensis* does not, however, encode homologs of this protein, although there are soluble multiheme *c*-type cytochromes that are expressed at high levels such as the tetraheme proteins Kustc1170 and Kuste2854. The latter was recently characterized in detail and found to exchange electrons with HZS.^23^ However, there is no a priori need for the redox partner of KsHAO to be a multiheme protein. As a typical trimeric HAO-like octaheme cytochrome *c*, KsHAO possesses a 24-heme electron relay system to transport electrons between the active site heme 4 of each monomer and electron-transfer sites on the surface of the protein.^24^ This system can store multiple electrons abstracted from a substrate molecule, so that they may be transferred to single heme proteins successively, as confirmed by the activity of KsHAO with single electron acceptors such as Kustc0563 and BtCytc.

On the surface of KsHAO, the predicted site for interactions with Kustc0563 between the protrusions on the tulip-bulb shaped trimer is essentially the same as that proposed for the interaction between the homologous NeHAO and its cognate redox partner, cytochrome c554, based on docking studies.^25^ Traditionally, these electron-transfer sites are believed to be located at heme 1 of each monomer in the protrusions on the trimer. Binding of a redox partner close to heme 1 was proposed to raise its redox potential, thus “opening a gate” allowing the transfer of an electron to heme 1 and from there to the redox partner.^26^ Moreover, in the related Kustc0457/Kustc0458 complex, the typical 24-heme electron transport system of HAO-like Kustc0458 is extended from its heme 1 onward with the hemes of the complex partner Kustc0457.^3^ Interestingly, however, in our simulations, heme 3, which in KsHAO lies at the surface of the protein at the bottom of the concave surface between the protrusions, is consistently predicted to be part of the interaction surface with Kustc0563. This opens up the possibility that in the interaction between KsHAO and Kustc0563, heme 3 is the outlet for electrons rather than heme 1.

## Materials and Methods

### Protein Preparation

Signal peptides were predicted in both the Kustc0562 and Kustc0563 protein sequences using the Signal 3.0 server, employing the hidden Markov model (HMM).^27^ The corresponding genes were then amplified without their signal peptides by PCR from *K. stuttgartiensis* genomic DNA using primers listed in Table S1. The amplified products were cloned into pUC19kan3 using NotI and XhoI restriction sites. This vector; which adds a tobacco etch virus (TEV) protease-cleavable, N-terminal His-tag to the coding region; uses the periplasmic signal sequence from a tetraheme cytochrome of *Shewanella oneidensis MR-1*(28) and is constitutively transcribed under a *lac* promoter. In addition, Kustc0563 was cloned into pUC19kan2a, which adds a noncleavable C-terminal His-tag. Cells of *S. oneidensis*MR-1 were then transformed with one of these vectors by electroporation, and the transformants were cultured in LB medium with 50 μg/mL kanamycin using 5 L nonbaffled flasks shaking at 90–95 rpm and 30 °C. When an OD600 of 0.6–0.8 was reached, shaking and temperature were reduced to 50–60 rpm and 20 °C, respectively, and incubation was continued for 60–70 h. The cells were then harvested by centrifugation at 6000 rpm in an F9-6X 1000 LEX rotor (Thermo Scientific, Darmstadt, Germany) for 10 min at 4 °C and suspended in a wash/lysis buffer (50 mM TrisCl pH 8.0, 300 mM NaCl, and 10 mM imidazole) at a 1:4 ratio of wet pellet weight to buffer volume. Cell lysis was performed by sonication in 100 mL portions on ice using a Branson W-450 sonifier (G. Heinemann, Schwäbisch Gmünd, Germany) at 50% amplitude with 0.5 s bursts and 10 minutes of total on time. The lysate was then cleared by ultracentrifugation at 160 000*g* for 45 min at 4 °C in a Ti-45 rotor (Beckman Coulter, Krefeld, Germany). The supernatant was then loaded onto Ni-NTA agarose (Qiagen, Hilden, Germany) or Ni-IDA Profinity beads (Bio-Rad laboratories GmbH, Munich, Germany) pre-equilibrated with wash buffer (50 mM TrisCl pH 8.0, 300 mM NaCl, and 10 mM imidazole). The flow-through was reloaded twice, after which the column was washed with 20 volumes of wash buffer. The proteins were then eluted in 50 mM TrisCl pH 8.0, 300 mM NaCl, and 250 mM imidazole. For the constructs with cleavable N-terminal His-tags, protein-containing eluate fractions were buffer-exchanged to TEV digest buffer (20 mM TrisCl pH 8.0, 150 mM NaCl, and 2 mM TCEP) before being concentrated to ≈1 mL using 3 kDa molecular-weight cutoff (MWCO) Amicon concentrators. TEV digestion was performed overnight at an  (protein)-to-TEV ratio of 100:1 in a cold room with gentle rotation. The next day, the Ni-NTA chromatography step was repeated to remove any uncleaved protein as well as the His-tagged TEV protease, collecting the flow-through that contained the cleaved protein. This material was again concentrated to ≈1 mL in a 3 kDa MWCO Amicon concentrator. Finally, size-exclusion chromatography (SEC) was performed for all constructs using a Superdex 75 (10/300 GL) column (GE Healthcare, Uppsala, Sweden). The main peak from the SEC was buffer-exchanged using Amicon concentrators to 25 mM HEPES/KOH pH 7.0 and 25 mM KCl and was concentrated to an . Protein purity was assessed by SDS–PAGE followed by heme and Coomassie staining. The identity of the purified proteins was confirmed by peptide mass fingerprinting. Gel slices of the respective protein bands from 15% SDS–PAGE were excised and digested by trypsin. Stable proteolytic fragments were analyzed by matrix-assisted laser desorption/ionization time-of-flight (MALDI-TOF) mass spectrometry on an Axima TOF2 Performance mass spectrometer (Shimadzu Biotech, Duisburg, Germany) using α-cyano-4-hydroxycinnamic acid as the matrix. Characteristic tryptic peptides were identified by MASCOT (Matrix Science Inc., MA, USA). Protein total mass analyses were performed under denaturing conditions by electrospray ionization time-of-flight (ESI-TOF) mass spectrometry on a maXis spectrometer (Bruker Daltonik GmbH, Bremen, Germany). Kustc0562 and Kustc0563 after cleavage of the N-terminal His-tag are designated Kustc0562nt and Kustc0563nt (nt = no tag), respectively. C-terminally His-tagged Kustc0563 will be designated Kustc0563CT (C-terminal tag). The protein sequences of the expression constructs are given in Figure S5.

### UV–Vis Spectroscopy

UV–vis spectra of Kustc0562nt and Kustc0563nt were measured in 200 μL quartz microcuvettes (path length = 1 cm, Hellma Analytics, Müllheim, Germany) at 0.5 nm bandwidth using a Jasco V-650 spectrophotometer (Jasco GmbH, Gross-Umstadt, Germany) equipped with a sample holder at 25 °C. Ferricyanide-oxidized samples of both proteins were diluted to 10 μM in a 20 mM potassium phosphate buffer pH 7.0, and spectra were recorded. A few grains of solid sodium dithionite or 100 mM imidazole were added as appropriate. Raw spectra were baseline-corrected using the Jasco software, and figures were generated using GraFit 7.0.0 (Erithacus Software Ltd, East Grinstead, U.K.).

### Enzyme Assays

The protein concentration of KsHAO was determined by the Bradford method^29^ using a dilution series of a bovine serum albumin standard solution (Thermo Scientific, Darmstadt, Germany) for calibration. HAO assays were performed essentially as described previously^8^ using Kustc0562nt, Kustc0563nt, and bovine cytochrome *c* (BtCytc, Sigma-Aldrich) as electron acceptors. All electron acceptor cytochromes were fully oxidized by incubation with 10 mM potassium ferricyanide for 30 min at room temperature followed by buffer exchange to a 20 mM pH 7.0 potassium phosphate buffer using a PD-10 desalting column (GE Healthcare, Uppsala, Sweden). The concentration of the electron acceptor cytochromes was assessed after full reduction with sodium dithionite based on the α-band absorbance using an extinction coefficient ε_550_ of 27.6 mM^–1^ cm^–1^ as reported for BtCytc.^30^ For the assays, 0.5 mL reaction mixtures were prepared, containing 0.6 μg of *K. stuttgartiensis* HAO (KsHAO) and 50 μM the appropriate electron acceptor cytochrome in 20 mM potassium phosphate buffer (pH 7.0) in polystyrene cuvettes. After 1 min of initial incubation at 37 °C, the reaction was started by adding the appropriate amount of hydroxylamine (1–100 μM final concentration), and the reduction of the electron acceptor was then followed by monitoring the increase in absorbance at 550 nm using Δε_550_ = 19.6 mM^–1^ cm^–1^ for BtCytc^30^ and Δε_550_ = 19.1 mM^–1^ cm^–1^ for Kustc0563nt. Averaged initial rates from three technical replicate series were fitted by applying the Michaelis–Menten equation. Kinetic parameters are reported in terms of the substrate (hydroxylamine) concentration.

### Redox Potential Determination

An optically transparent thin-layer electrochemical (OTTLE) cell was constructed and operated using a Keithley model 2450 source measure unit (Tektronix Inc., Beaverton, OR, USA) functioning as a potentiostat. To this end, thin (< 1 μm) gold electrode connections were produced on a 26 mm × 76 mm glass microscope slide by spray coating with gold paint (Glanzgold GG B 15/M, Heraeus, Hanau, Germany) and subsequent heating to 520 °C. Moreover, a silver/silver chloride reference electrode patch was prepared on the slide using Ag/AgCl ink (ALS Co., Ltd, Tokyo, Japan). A working electrode was prepared using a gold mesh (500 wires/inch, 60% open area, 10 μm thickness; Goodfellow Ltd., Huntingdon, U.K.) that was modified by incubation for >60 min in a solution containing 20 mM 4,4′-dithiodipyridine (DTP) in 160 mM pH 8.0 TrisCl and 20% (v/v) ethanol. For spectroelectrochemical measurements, 18 μL of the protein solution in 100 mM KCl and 10 mM pH 7.0 MOPS/KOH was mixed with 2 μL of a small-molecule mediator mix (1 mM each of potassium ferricyanide, *p*-benzoquinone, 2,5-dimethyl-*p*-benzoquinone, 1,2-naphtoquinone, phenazine methosulfate, 1,4-napthoquinone, phenazine ethosulfate, 5-hydroxy-1,4-napthoquinone, 2-methyl-1,4-napthoquinone, 2,5-dihydroxy-*p*-benzoquinone, 2-hydroxy-1,4-napthoquinone, anthraquinone, sodium anthraquinone-2-sulphonate, benzyl viologen, and methyl viologen). The protein solution was then pipetted onto the working electrode that was placed on the gold electrodes on the slide and covered with a standard 22 mm × 22 mm glass microscope cover slip. After sealing with parafilm, the cell was mounted in the beam of a Jasco V-760 spectrophotometer (Jasco GmbH, Gross-Umstadt, Germany). The Keithley source meter was used to set potentials and measure currents and also controlled data acquisition by the spectrophotometer using a custom-built interface. Samples were prepoised at −300 mV for 10 min and then oxidized to +300 mV, after which they were reduced back again to −300 mV in 20–60 mV steps. UV–vis spectra were recorded using wavelengths ranging from 350 to 700 nm (90 s/spectrum at 400 nm/min). Raw spectra were baseline-corrected by setting the absorption at 700 nm to zero. Absorbance values at 420 nm (the Soret band) were fitted to a Nernstian function using nonlinear least-squares minimization in Microsoft Excel to determine the midpoint potential *E*_m_:
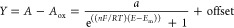
where *A* is the absorbance, *A*_ox_ is the absorbance of the fully oxidized state, *a* is the amplitude, *E* is the applied potential, *E*_m_ is the midpoint potential, and *n* is the number of electrons (*n* = 1). For each protein, data from two individual experiments were fitted together. To remove hysteresis effects likely resulting from protein denaturation, the oxidative and reductive limbs of each data series were normalized individually. Spectra for one of each of the data series for Kustc0562 and Kustc0563 are given in Supporting Information Figure S3. The Faraday constant *F* was taken to be 96 485.34 J V^–1^ mol^–1^, the temperature *T* was 293 K, and the ideal gas constant *R* was taken to be 8.3145 J mol^–1^ K^–1^. To correct the potential against the SHE, the voltage between the Ag/AgCl patch in a drop of 10 mM MOPS/KOH (pH 7.0) and 100 mM KCl and a commercial Ag/AgCl/4 M KCl reference electrode (Pine Research Instrumentation, Durham, NC, USA; *E*° = +200 mV vs SHE) was measured.

### Crystallization, Data Collection, and Structure Solution

Kustc0562nt was crystallized in 1 + 1 μL hanging drop setups equilibrating against 0.2 M NaCl, 30% (w/v) PEG 8000, 0.1 M imidazole pH 8.0, and 0.277 mM cyclohexylethanoyl-*N*-hydroxyethylglucamide (C-HEGA-8). Flattened needles of up to 400 μm in length grew within 2 days at room temperature. These were cryoprotected by soaking in reservoir solution supplemented with 25% (v/v) ethylene glycol prior to flash-cooling in liquid nitrogen. A 3.1 Å resolution data set was collected on a Rigaku MicroMax 007HF rotating anode equipped with a MAR345 image plate. Kustc0563CT was crystallized in 1 + 1 μL hanging drop setups equilibrating against 15% (w/v) PEG 6000, 0.5 M LiCl, and 0.1 M sodium cacodylate (pH 6.5), with 0.2 μL of 80 mM CHAPS added to the drop. These were cryoprotected by soaking in mother liquor supplemented with 20–25% (v/v) ethylene glycol and flash-cooled in liquid nitrogen. A high redundancy SAD dataset was collected at 100 K and 1.7433 Å wavelength at beamline X10SA at the Swiss Light Source (SLS) of the Paul Scherrer Institute (Villigen, Switzerland). Bipyramidally shaped crystals of KustC0563nt were obtained from 0.2 M zinc acetate and 20% (w/v) PEG 3350 in 96-well sitting drop vapor diffusion setups with a drop size of 100 nl + 100 nl, with the concentration of protein being such that . Crystals of about 150 × 100 μm^2^ grew in 4–6 days and were flash-frozen in liquid nitrogen using 20% (v/v) ethylene glycol in the reservoir solution as a cryoprotectant. High-resolution diffraction data were collected from these crystals at beamline X10SA at the SLS of the Paul Scherrer Institute (Villigen, Switzerland) at 100 K. All diffraction data were processed with XDS,^31^ and data statistics are reported in Table S2.

### Structure Determination and Refinement

The Kustc0563CT diffraction data were phased by single-wavelength anomalous diffraction (SAD) using phenix.autosol,^32,33^ which identified six iron sites indicating six molecules in the asymmetric unit. An initial structure was built automatically, which was completed and refined by iterative cycles of rebuilding in COOT^34^ and refinement by REFMAC5.^35^ The Kustc0563nt data were phased by molecular replacement using MOLREP^36^ using a monomer from the KustC0563CT structure as the search model. The final model was refined using REFMAC5.^35^ The Kustc0562nt data were phased by molecular replacement using Phaser,^37,38^ using the Kustc0563nt structure as the search model, and refined using REFMAC5^35^ and phenix.refine.^32^

### Monte Carlo Interaction Site Mapping

Structures were prepared by adding hydrogen and assigning charges with the PDB2PQR server^39−41^ using the PARSE force field^42^ and PROPKA^43^ to assign protonation states at pH 7.0. For *c*-type heme moieties, parameters were adapted from the PARSE force field as in the work of Akram et al.^10^ Electrostatic potentials were calculated using APBS 3.0^44^ assuming a 20 mM potassium phosphate buffer, and Monte Carlo mapping was done with MCMAP 1.0,^16^ performing 1 000 000 runs of 25 000 steps each at a temperature of 300 K and using a maximum center-of-mass distance of 500 Å. Results were collated and visualized using the PyCoALA toolbox. The calculations were performed with various combinations of heme states: all ferric, all ferrous, all ferrous on KsHAO but the heme in Kustc0563 ferric, or all hemes in the ferric state but with an electron on either heme 3 or heme 1 of KsHAO. All calculations resulted in essentially the same interaction surface. However, because the calculation with all hemes in the ferrous state identified the most residues in the interaction surfaces, the results from this calculation are shown in Figure 3.

## Supporting Information Available

Figures including transcription patterns of Kustc0562/63, UV–vis spectra and raw spectroelectrochemistry data of Kustc0562nt and Kustc053nt, Michaelis–Menten kinetic data, and protein sequences of the expression constructs used in this study; tables including protein crystal structures and crystallographic data deposited in the protein data bank under entry codes 5mxy and 7o38 (PDF)

## Abbreviations

Tris: 2-amino-2-hydroxymethyl-propane-1,3-diol
TCEP: tris(2-carboxyethyl)phosphine
k_B_: Boltzmann’s constant
PEG: polyethylene glycol
MOPS: 3-morpholinopropane-1-sulfonic acid
NTA: nitrilotriacetic acid
IDA: iminodiacetic acid
and SDS–PAGE: sodium dodecyl sulfate polyacrylamide gel electrophoresis
