# Supplementary material for: Specificity of Small c-Type Cytochromes in Anaerobic Ammonium Oxidation
Source: ACS Omega. 2021 Aug 9;6(33):21457–64. doi: 10.1021/acsomega.1c02275 (PMC8388095; doi:10.1021/acsomega.1c02275)
Supplement: Supplementary file 1 — ao1c02275_si_001.pdf [file ao1c02275_si_001.pdf]

Supporting information for:

Specificity of small c-type cytochromes in anaerobic ammonium oxidation

Mohd. Akram, Josephine Bock, Andreas Dietl* and Thomas R.M. Barends*

Max Planck Institute for Medical Research, Jahnstrasse 29, D-69120 Heidelberg, Germany

*Corresponding authors: Andreas.Dietl@mr.mpg.de, Thomas.Barends@mr.mpg.de

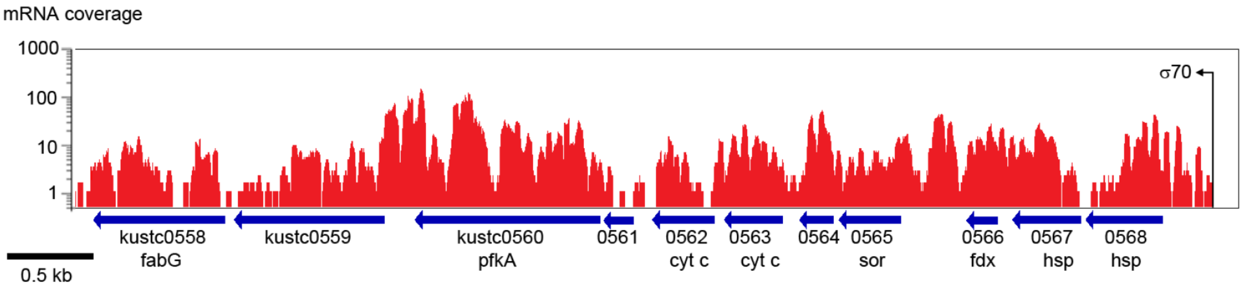

Supporting Figure S1. Transcription patterns of Kustc0562/63 and their neighbouring genes. fabG: 3-oxoacyl-[acyl-carrier-protein] reductase; pfkA: 6-phosphofructokinase; cyt c: class I cytochrome c; sor: superoxide reductase; hsp: small heat shock protein. mRNA reads from a metatranscriptome dataset (Gene expression omnibus GSE15408, SRR037687) were mapped against the *K. stuttgartiensis* reference genome. The graph shows the mRNA read coverage plotted on a logarithmic scale against the length of the genomic DNA and was prepared using the Integrative Genomics Viewer[37]. The bent arrow indicates a σ70 promotor site predicted using BPROM[38]. The genes Kustc0562 and Kustc0563 show comparable transcription levels to each other and to constitutively expressed proteins.

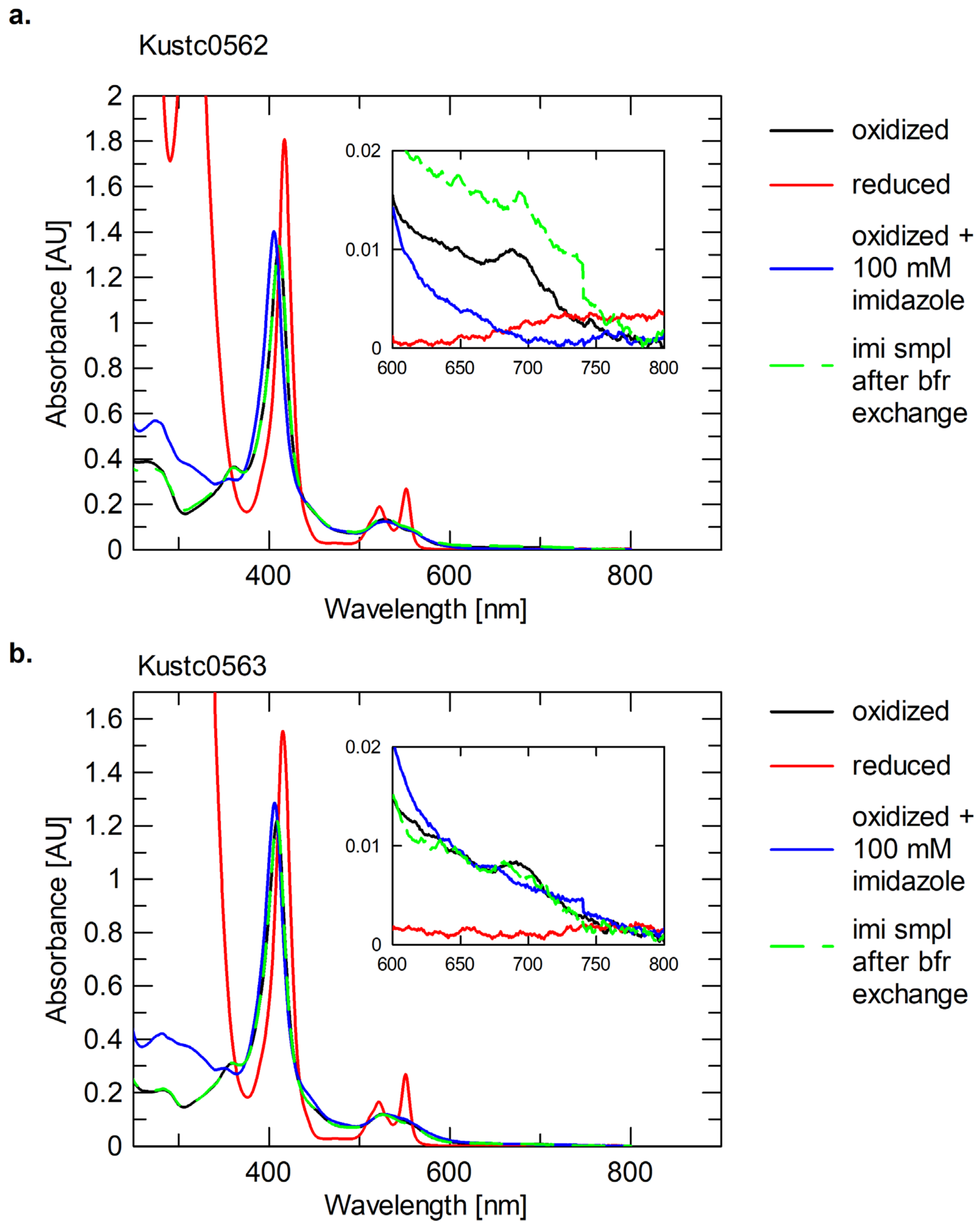

Supporting Figure S2. UV-Vis spectra of Kustc0562nt (a) and Kustc053nt (b).

The UV-Vis spectra of 10 µM Kustc0562 or Kustc0563 (untagged) were measured in 20 mM potassium phosphate buffer, pH 7.0.

(a) The spectrum of oxidized Kustc0562 (black) shows a Soret band maximum at 411 nm and a broad band at 528 nm. The spectrum of the same sample after full reduction with sodium dithionite (red) shows a red-shifted Soret band to 417 nm as well as α- and β-bands at 551 nm and 522 nm, respectively. (b) The spectrum of oxidized Kustc0563 (black) shows a Soret band maximum at 409 nm and a broad band at 525 nm. The spectrum of the same sample after full reduction with sodium dithionite (red) shows a red-shifted Soret band to 415 nm as well as α- and β-bands at 551 nm and 522 nm, respectively. Samples of both oxidized Kustc0562 and Kustc0563 supplemented with 100 mM imidazole (blue) show a blue-shifted Soret band maximum at 406 nm. Inset: close-up of the charge-transfer band region. Both oxidized Kustc0562 and Kustc0563 show a weak charge-transfer band at around 690 nm, indicative of methionine coordination to the heme iron. This band disappears upon reduction or ligand-exchange to imidazole. After buffer exchange of the imidazole-treated samples, the spectral characteristics of the oxidized proteins are restored (green dashed line). All spectra were baseline corrected. The spectra of the buffer-exchanged samples were scaled to the spectra of the oxidized proteins based on the absorbance of the Soret band maxima (411 nm and 409 nm for Kustc0562 and Kustc0563, respectively).

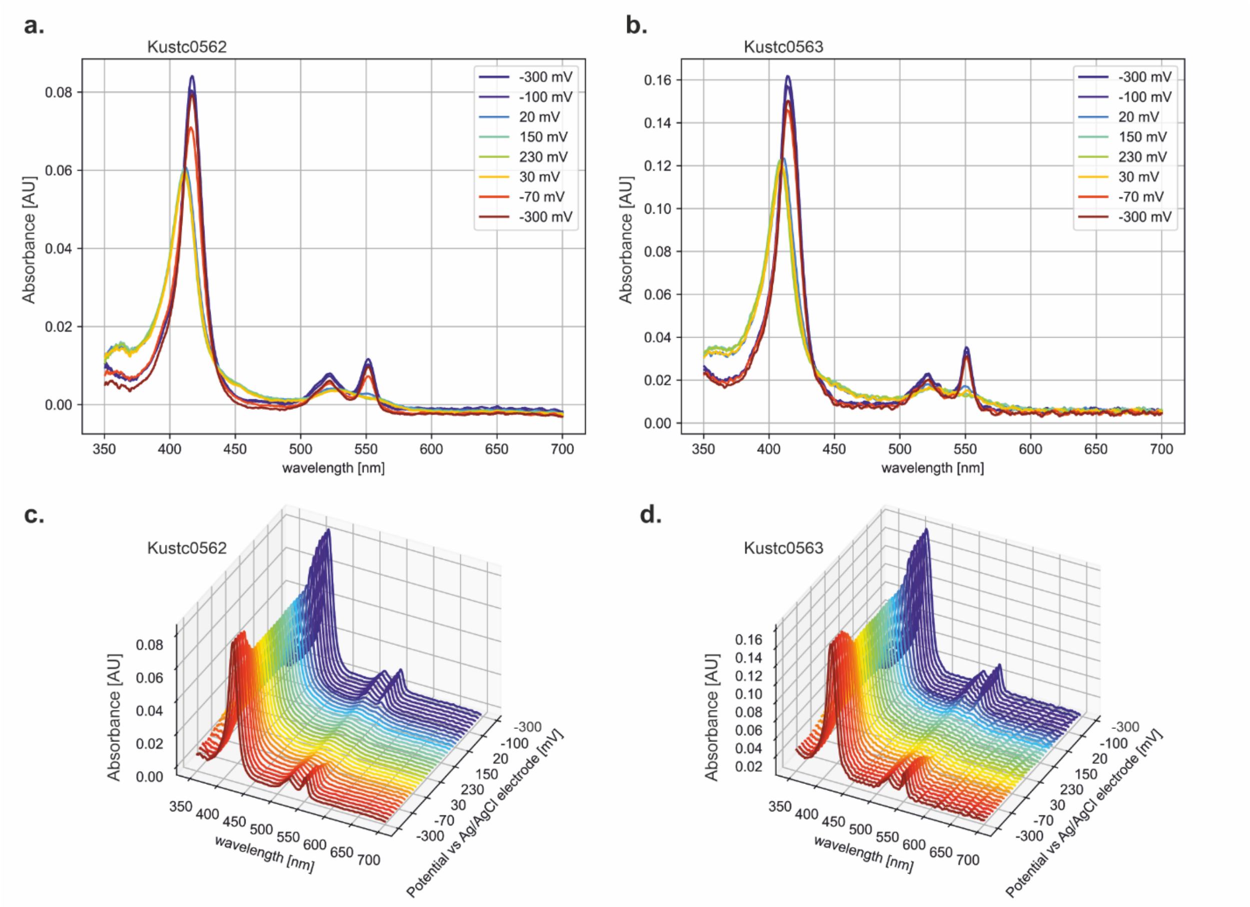

Supporting Figure S3. Raw spectroelectrochemistry data for Kustc0562nt (a,c) and Kustc0563nt (b,d). The top panels show selected spectra from the 35 potentials used, the bottom panels show the full family of curves for one each of the two data series used in the redox potential determination for each of the two proteins. The redox potential is given against an Ag/AgCl electrode patch in the OTTLE cell using 100 mM KCl in the buffer.

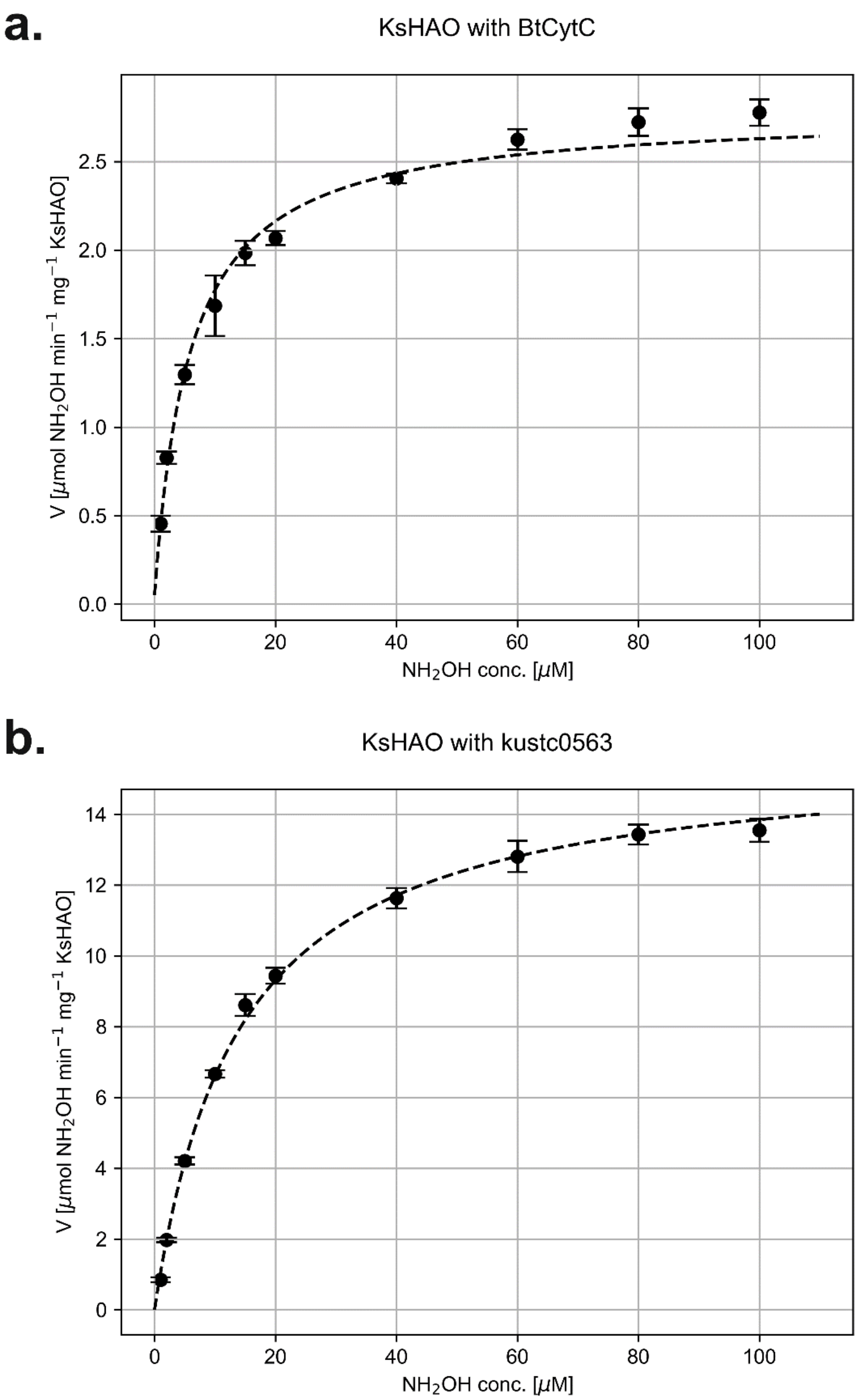

Supporting Figure S4. Michaelis-Menten kinetic data for hydroxylamine oxidation by KsHAO using BtCytc (a) or Kustc0563nt (b) as electron acceptors. The assays (0.5 mL) were performed in polystyrene cuvettes thermostatted at 37 °C using 20 mM potassium phosphate buffer, pH 7.0. Each reaction contained 0.6 µg KsHAO, 50 µM of either BtCytc or Kustc0563 and variable concentrations of hydroxylamine (1-100 µM). The average values ± standard deviation from three technical replicates were plotted (black dots) and fitted to the Michaelis-Menten equation (dashed line).

Kustc0563CT (pUC19Kan2a_Kustc0563)

AANIDGMKLYLQHCKTCHGVDGNPTDLGEGLGARKFADAEWQAKTSDERIIEQINEGTPEMMMPFKEKLTPEEVKALVPVVRGFKK**L**
EHHHHHH
94 Amino acids
mW = 10,625.09 Da pI = 6.11 (estimated)
ext= 7115.00 M^{-1}cm^{-1} abs=0.670 (1g/l), assuming ALL Cys residues appear as half cystines
ext= 6990.00 M^{-1}cm^{-1} abs=0.658 (1g/l), assuming NO Cys residues appear as half cystines
Negative (Asp + Glu): 15 Positive (Arg + Lys): 12

Kustc0563nt (pUC19Kan3_Kustc0563)

before TEV cleavage:
ADHHHHHHGSGSENLYFQGAAANIDGMKLYLQHCKTCHGVDGNPTDLGEGLGARKFADAEWQAKTSDERIIEQINEGTPEMMMPF
KEKLTPEEVKALVPVVRGFKK
106 Amino acids
mW = 11,780.24 Da pI = 6.04 (estimated)
ext= 8605.00 M^{-1}cm^{-1} abs=0.730 (1g/l), assuming ALL Cys residues appear as half cystines
ext= 8480.00 M^{-1}cm^{-1} abs=0.720 (1g/l), assuming NO Cys residues appear as half cystines
Negative (Asp + Glu): 16 Positive (Arg + Lys): 12

after TEV cleavage:
GAAANIDGMKLYLQHCKTCHGVDGNPTDLGEGLGARKFADAEWQAKTSDERIIEQINEGTPEMMMPFKEKLTPEEVKALVPVVRGFK
K
88 Amino acids
mW = 9,688.11 Da pI = 6.04 (estimated)
ext= 7115.00 M^{-1}cm^{-1} abs=0.734 (1g/l), assuming ALL Cys residues appear as half cystines
ext= 6990.00 M^{-1}cm^{-1} abs=0.722 (1g/l), assuming NO Cys residues appear as half cystines
Negative (Asp + Glu): 14 Positive (Arg + Lys): 12

Kustc0562nt (pUC19Kan3_Kustc0562)

before TEV cleavage:
AADHHHHHHGSGSENLYFQGAAAEEKAIDARNLFEYHCAKCHGLTGEANKRGKALKAPDLCDPGWQNSKTDKEILYSITNGKNKM
PAWNERLTPEEIEALARYVRKLSKKQR
112 Amino acids
mW = 12,650.15 Da pI = 6.28 (estimated)
ext= 17147.00 M^{-1}cm^{-1} abs=1.355 (1g/l), assuming ALL Cys residues appear as half cystines
ext= 16960.00 M^{-1}cm^{-1} abs=1.341 (1g/l), assuming NO Cys residues appear as half cystines
Negative (Asp + Glu): 15 Positive (Arg + Lys): 18

after TEV cleavage:
GAAAEEKAIDARNLFEYHCAKCHGLTGEANKRGKALKAPDLCDPGWQNSKTDKEILYSITNGKNKMPAWNERLTPEEIEALARYVRK
LSKKQR
93 Amino acids
mW = 10,486.94 Da pI = 6.32 (estimated)
ext= 15657.00 M^{-1}cm^{-1} abs=1.493 (1g/l), assuming ALL Cys residues appear as half cystines
ext= 15470.00 M^{-1}cm^{-1} abs=1.475 (1g/l), assuming NO Cys residues appear as half cystines
Negative (Asp + Glu): 13 Positive (Arg + Lys): 18

Supporting Figure S5. Protein sequences of the expression constructs used in this study
The sequences are shown after the cleavage of the N-terminal signal peptide. Amino acids that are not part of the original protein sequence are shown in bold, hexa-histidine tags and tobacco etch virus (TEV) protease cleavage sites are underlined.

Supporting Table S1. PCR Primer sequences

Name	Sequence 5'→ 3'	Length (nt)	Comments
C0562_F1	CCTTTTCTTGCGGCCGCGGAAGAAAAGGCCATAGACG	37	Amplification of kustc0562, for cloning into pUC19kan3, NotI site
C0563_F1	TCTGTTTCAGCGGCCGCGAACATTGATGGT	30	Amplification of kustc0563, for cloning into pUC19kan2a and pUC19kan3, NotI site
C0563_R1	CAGCACCTCGAGCTTTTTAAACCCTCTGA	29	Amplification of kustc0563, for cloning into pUC19kan2a, XhoI site
C0562_R2	CAATGGCTCGAGTCACCGTTGCTTTTTTGATAATTTCC	38	Amplification of kustc0562, for cloning into pUC19kan3, XhoI site, stop codon
C0563_R2	TTTCAGCTCGAGTTACTTTTTAAACCCTCTGAC	33	Amplification of kustc0563, for cloning into pUC19kan3, XhoI site, stop codon

Supporting Table S2. Crystallographic data collection and refinement statistics.
Values for the highest resolution shell are given in parentheses.

**Data set**	**Kustc0562nt (pdb entry 7O38)**	**Kustc0563CT Fe-SAD**	**Kustc0563nt (pdb entry 5MXY)**
Space group	$P6_4$	$P2_12_12_1$	$P4_32_12_1$
Unit cell dimensions			
a, *b*, *c* [Å]	101.7, 101.6, 28.3	84.8, 49.2, 253.3	47.8, 47.8, 99.9
α, β, γ [°]	90, 90, 120	90, 90, 90	90, 90, 90
Wavelength [Å]	1.5418	1.736	1.000
Resolution range [Å]	40.0-3.0 (3.1-3.0)	40.0-2.7 (2.8-2.7)	50.0-1.9 (2.0-1.9)
No. reflections	8,066	378,921	65,768
No. unique reflections	3,395	32,168	9,541
Completeness [%]	95.8 (90.6)	99.3 (94.6)	97.7 (98.7)
Redundancy	2.4 (2.1)	11.8 (5.6)	6.9 (6.9)
I/σI	6.5 (2.7)	15.5 (2.1)	12.2 (1.5)
R_{merge} [%]	3.7 (16.6)	10.3 (54.3)	8.1 (278.6)
R_{meas} [%]	4.1 (17.4)	10.7 (59.8)	8.8 (301.1)
$CC_{1/2}$	0.973 (0.745)	0.998 (0.866)	0.999 (0.823)
CC*	0.993 (0.924)	0.999 (0.963)	0.999 (0.950)
**Refinement**			
R_{work}/R_{free}	0.191/0.248		0.198/0.229
No. residues/atoms	80/646		83/773
No. ligands/atoms	1 heme/43, 1 imidazole/5, 1 chloride, 8 waters/8		1 heme/43, 68 waters/68
Overall B-factor [$Å^2$]	22.2		49.1
RMSD bonds	0.013		0.009
RMSD angles	1.573		2.29
No. residues in region of Ramachandran plot			
Most favored	97.5		93.8
Allowed	2.5		6.2
Disallowed	0.0		0.0
